# RNA splicing in cancer cell death regulation: shedding light on the molecular mechanisms and potential clinical applications

**DOI:** 10.1038/s41419-026-08687-0

**Published:** 2026-05-22

**Authors:** Minghui Song, Yaru Wang, Haiyun Zhang, Xinyu Ji, Shuqing Sun, Zhuting Fang, Jie Ying, Qian Li, Jianxiang Chen

**Affiliations:** 1 School of Pharmacy and Department of Hepatology, the Affiliated Hospital of Hangzhou Normal University, Hangzhou Normal University, Hangzhou, PR China; 2 Department of Oncology and Vascular Interventional Therapy, Clinical Oncology School of Fujian Medical University, Fujian Cancer Hospital, NHC Key Laboratory of Cancer Metabolism, Fuzhou, China; 3 Department of Gastroenterology, Nanjing Jiangbei Hospital, Yangzhou University College of Clinical Medicine, Nanjing, China; 4 Zhejiang Provincial Key Laboratory of Anti-Cancer Chinese Medicines and Natural Medicines, Hangzhou Normal University, Hangzhou, PR China

**Keywords:** Oncogenes, Cancer

## Abstract

Alternative splicing (AS) is a ubiquitous posttranscriptional regulatory mechanism in eukaryotes through which proteins with various structures and functions are produced from a single pre-mRNA. It is performed by the spliceosome and regulated by both cis-acting elements and trans-acting splicing factors. The common modes of regulatory cell death (RCD) include apoptosis, necroptosis, autophagy, pyroptosis and ferroptosis, and dysregulation of these cell death modes can lead to tumorigenesis. The abnormal expression of spliceosome components and splicing factors, or aberrant splicing events, modulates RCD and contributes to the occurrence and progression of cancer. This review focuses on the roles of AS in tumour RCD and the underlying mechanism through which AS modulates different modes of RCD. Moreover, we summarise some potential clinical applications of targeting AS–RCD axes. This review offers systematic and mechanistic insights into tumorigenesis from the perspectives of AS and RCD, and the ultimate objective is to translate these findings into targeted therapies for cancer treatment.

## Facts

- Evading cell death is a hallmark of tumour cells.
- Having a thorough understanding of the various pathways of cell death contributes to the development of preventive measures and targeted treatments for cancer.
- AS is an important post-transcriptional regulatory mechanism in eukaryotes, aberrant AS can lead to the occurrence and metastasis of cancer.
- Converting the AS targets in the RCD pathway to clinical applications may potentially address some of the challenges in cancer treatment encountered in clinical settings.

## Open questions

- Is understanding the regulatory mechanism of various RCD beneficial for the treatment of cancer?
- How AS regulates various forms of cell death in cancer?
- Which small molecule inhibitors and SSOs target AS-RCD axes are currently available?

## Introduction

Cell death is a fundamental physiological process in all organisms, and cancer is a heterogeneous disease caused by the dysregulation of cell death [1]. Cell death is mainly divided into accidental cell death and regulatory cell death (RCD), and RCD is characterised mainly by apoptosis, pyroptosis, ferroptosis, necroptosis and autophagy [2]. In contrast to accidental cell death, RCD is controlled by specific signal transduction pathways, which can be regulated by genetic signals or pharmacological interventions [3].

**Fig. 1:**
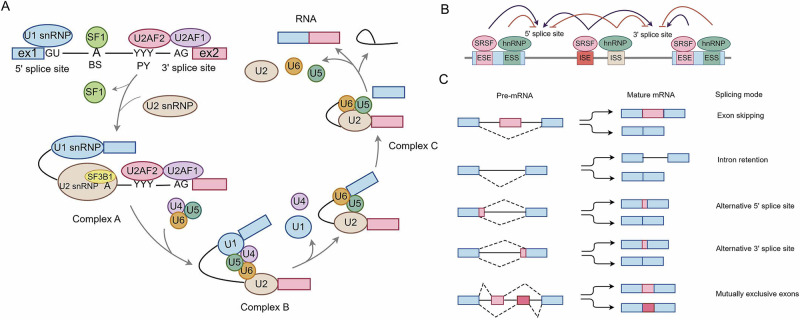
Pre-mRNA splicing, its regulatory mechanism and the modes of alternative splicing. **A** U1 snRNP binds to the 5’ splice site, and U2AF1 recognises the 3’ acceptor splice site; U2AF2 binds to the adjacent poly-pyrimidine tract, and the auxiliary factor splicing factor 1 (SF1) binds to the branch site (BS). Subsequently, U2 snRNP replaces SF1 and binds to BS, during which SF3B1 promotes the recognition of BS. Then, the U4/U5/U6 snRNP complex binds to the 5’ splice site to form complex B. Finally, U1 and U4 snRNPs are dissociated from the system, and the spliceosome catalyses two transesterification reactions, releasing the circular intron and joining the exons together. **B** Generally, SRSFs interact with splicing enhancers to promote the recognition of splice sites; hnRNPs interact with splicing silencers to inhibit the recognition of splice sites. **C** Different types of alternative splicing. Blue indicates exon that always be included in the mature mRNA; light pink and dark pink indicate exons that can be included or excluded in the mature mRNA. BS branch site, PY polypyrimidine region, ESE exonic splicing enhancer, ISE intronic splicing enhancer, ESS exonic splicing silencer, ISS intronic splicing silencer.

Unlike prokaryotic genes, eukaryotic genes exhibit a structure where the protein-coding exons are interrupted by introns. RNA splicing is the process through which introns are excluded from the precursor messenger RNA (pre-mRNA) and exons are connected to generate mature mRNA [4]. This process involves multiple steps (Fig. 1A) [5] and carried out by spliceosome, which includes five small nuclear ribonucleoproteins (U1, U2, U4, U5, and U6, snRNPs) [6]. First, U1 snRNP recognises the 5’ splice site, U2AF1 recognises the 3’ acceptor splice site; U2AF2 interacts with the adjacent polypyrimidine region, and auxiliary factor splicing factor 1 (SF1) binds to the branch site (BS). Next, U2 snRNP replaces SF1 and interacts with BS, forming complex A, in which SF3B1 promotes the recognition of BS. Subsequently, the U4/U5/U6 snRNP complex recognises the 5’ splice site to form complex B. Then, U1 and U4 snRNPs separates from the system and the spliceosome catalyses two transesterification reactions to release the lasso-like intron and join the exons together [7].

Alternative splicing (AS) is a mechanism to generate multiple mature mRNAs with distinct functions from a single gene through selecting different splice sites. It is regulated by both trans-acting factors and cis-acting elements, the cis-acting elements on pre-mRNA include exonic splicing enhancer (ESE), exonic splicing silencer (ESS), intronic splicing enhancer (ISE) and intronic splicing silencer (ISS) [8]. The serine/arginine-rich splicing factors (SRSFs) and heterogeneous nuclear ribonucleoproteins (hnRNPs) are two important splicing factor families that act as trans-acting factors. The activity of splicing factors and cis-acting elements is context-dependent, typically, SRSFs bind to splicing enhancers to promote splicing and hnRNPs bind to splicing silencers to inhibit splice site selection (Fig. 1B) [9]. The different modes of AS include exon skipping, alternative 5’ splice site, alternative 3’ splice site, intron retention and mutually exclusive exons (Fig. 1C).

Most cancers exhibit splicing abnormalities and tumours have more AS events than normal tissues [10], these abnormal events contribute to many hallmarks of cancer [11]. The spliced isoforms related to cancer have been shown to regulate nearly every aspect of cancer, including tumour progression [12, 13], angiogenesis [14], therapeutic resistance [15] and tumour microenvironment [16]. Splicing abnormalities in cancer can be caused by cis-acting mutations on pre-mRNA, which disrupt normal splice sites [17]. Furthermore, alternations of the activity and expression of spliceosome components and splicing factors also lead to aberrant AS in cancer. For example, *SF3B1*, *U2AF1* and *SRSF2* are frequently mutated and these mutations affect their binding to pre-mRNA [18].

Mutations and changes in the expression levels of spliceosome components and splicing factors, or aberrant splicing events, modulate RCD, promote the occurrence and progression of cancer and are potential targets for tumour treatment [19, 20]. The roles of SRSFs and hnRNPs in the regulation of apoptosis in cancer have been summarised. However, in recent years, there have been new advances in this field. Moreover, an increasing number of studies have reported that splicing factors regulate other RCDs, such as necroptosis, ferroptosis, pyroptosis, and autophagy. In this review, we summarise the role of AS in the regulation of RCD in cancer and its specific mechanisms (Table 1). Moreover, the potential clinical applications of AS–RCD axis-based therapeutics are discussed.

**Table 1:** Representative splicing factors and their regulatory roles in cell death pathways within different cancers.

RCD	Protein	Splice variant	Variant function	Splicing factor	Tumour type	References
Apoptosis	fas receptor	mFas, sFas	mFas (+), sFas (-)	SRSF6, PTBP1	Breast cancer, Nasopharyngeal cancer, HCC, CRC	[18–22]
	cFLIP	c-FLIP (S),c-FLIP (L)	c-FLIP (S) (-), c-FLIP (L) (+)	hnRNPA2	Lung cancer	[23, 24]
	caspase 8	Casp8L, Casp8-ΔE4, Casp8-ΔE7	(+)		T cell leukaemia	[26]
	caspase 2	Casp-2L, Casp-2S	Casp-2L (+), Casp-2S (-)	SRSF9	Cervical cancer	[28, 29]
	caspase 9	caspase-9a, caspase-9b	caspase-9a (+),caspase-9b (-)	hnRNPL, DDX17, SFPQ, P54, SRSF2	Lung cancer, EOC, PDAC	[30–33]
	Bcl-x	Bcl-xS, Bcl-xL	Bcl-xS (+), Bcl-xL (-)	RBM25, hnRNPA2/B1	Lung cancer, CRC, Oesophageal cancer	[34–36]
	BCL2L12	BCL2L12-L, BCL2L12-S	BCL2L12-L (-), BCL2L12-S (+)	BUD31	Ovarian cancer	[38]
	Mcl-1	Mcl-1L, Mcl-1S	Mcl-1L (-), Mcl-1S (+)	hnRNPF/H1/K, SRSF1	Breast cancer, ESCC	[49–51]
	Bim	Bim (S), Bim (L), Bim (EL)	Bim (S) (+), Bim (L) (+), Bim (EL) (+)	PTBP1, hnRNP C, SRSF2	RCC, CML	[45–47]
	Bax	Bax L, Bax S, Bax∆2	Bax L (+),Bax S (-), Bax∆2 (+)	PQBP1	Ovarian cancer, CRC	[40–42]
	survivin	Survivin, survivin-2α, survivin-2B, survivin-3B, survivin-δ Ex3	Survivin, survivin-δ Ex3, survivin-3β (-), survivin-2α, survivin-2β (+)	Sam68	Breast cancer, RCC, Cervical cancer, PDAC	[53–55]
Necrosis	MLKL	MLKL1, MLKL0	MLKL1 (+), MLKL0 (-)		CRC, lung cancer	[61]
	NF-YA	NF-YAx	NF-YAx (+)			[65]
Pyroptosis	STAT3	STAT3α, STAT3β	STAT3β (+)		ESCC	[70, 71]
	GSDMB	GSDMB 1-5	GSDMB3/4 (+), GSDMB1/2/5 (-)			[72, 74, 75]
Ferroptosis	SLC7A11	SLC7A11-L/S	SLC7A11-L (+), S (-)	SFPQ, NKAP, FMRP, hnRNPM	GBM, Breast cancer	[81, [82]
	TFRC	TFRC-S	TFRC-S (-)	RBFOX2	EC	[84]
	SAT1	SAT1-S	SAT1-S (+)	RNF113A	Lung cancer	[86]
	p73	p73α, p73β	p73β (+)		Lung cancer	[88]
	PCLAF	PCLAF-S/L	PCLAF-L (-)	SRSF2	HCC	[89]
	AR	AR	AR	HNRNPF, SF3B3	CRPC	[90–93]
	GPX4			SRSF9	CRC	[94]
Autophagy	mTOR	mTORα, mTORβ	mTORα (-)	SF3B3	Breast cancer, CRC, HCC, Cervical cancer	[101]
	BECN1	BECN1-α, BECN1-β, BECN1-γ	BECN1-α (+), BECN1-β (-)	SRSF3	Cervical cancer, CRC, Breast cancer, Ovarian cancer	[102–104]
	Atg16L	ATG16L1α, ATG16L1β	ATG16L1β (-)		NSCLC	[105, 106]
	Atg5	variants 2, 3	variants 2, 3 (+)		Prostate cancer	[107]
	Bcl-x	Bcl-xL, Bcl-xS	Bcl-xL (-)	SRSF1	Lung cancer	[108]
	BNIP3L	BNIP3L-F, BNIP3L-Δ1	BNIP3L-F (+), BNIP3L-Δ1 (-)	CTCF, BORIS, SRSF6	Breast cancer	[109]
	TFEB	TFEBP-L, TFEB-S	TFEBP-L (+)		AML	[110, 111]

+:promotes; -: inhibits. *CRC* colorectal cancer, *HCC* hepatocellular carcinoma, *EOC* epithelial ovarian cancer, *PDAC* pancreatic ductal carcinoma *ESCC* oesophageal squamous cell carcinoma, *RCC* renal cell carcinoma, *CML* chronic myelogenous leukaemia, *PDAC* pancreatic ductal cancer, *GBM* glioblastoma, *EC* endometrial cancer, *CRPC* castration-resistant prostate cancer, *NSCLC* non-small cell lung cancer; *AML* acute myeloid leukaemia.

## AS involved in regulating apoptosis

Apoptosis is a normal phenomenon in various organisms and plays an important role in eliminating dangerous cells. It is one of the natural ways for organisms to avoid the occurrence and progression of tumours [21]. The apoptosis pathway includes mainly the extrinsic pathway mediated by death receptors and the intrinsic pathway mediated by mitochondria. In the extrinsic pathway, after binding to its ligand, Fas death receptors oligomerize and recruit adaptor proteins to form death-inducing signalling complexes, which activate procaspase 8/10 [22]. Caspase 8/10 hydrolyses and activates caspases 3 and 7, initiating apoptosis [23]. cFLIP is an important regulator of death receptors and inhibits apoptosis by competing with procaspase-8 for binding to the adaptor protein FADD [24]. Moreover, caspase-2 activated by the PIDDosome or caspase-8 cleaves Bid, causing the release of cytochrome C and promoting apoptosis [25].

**Fig. 2:**
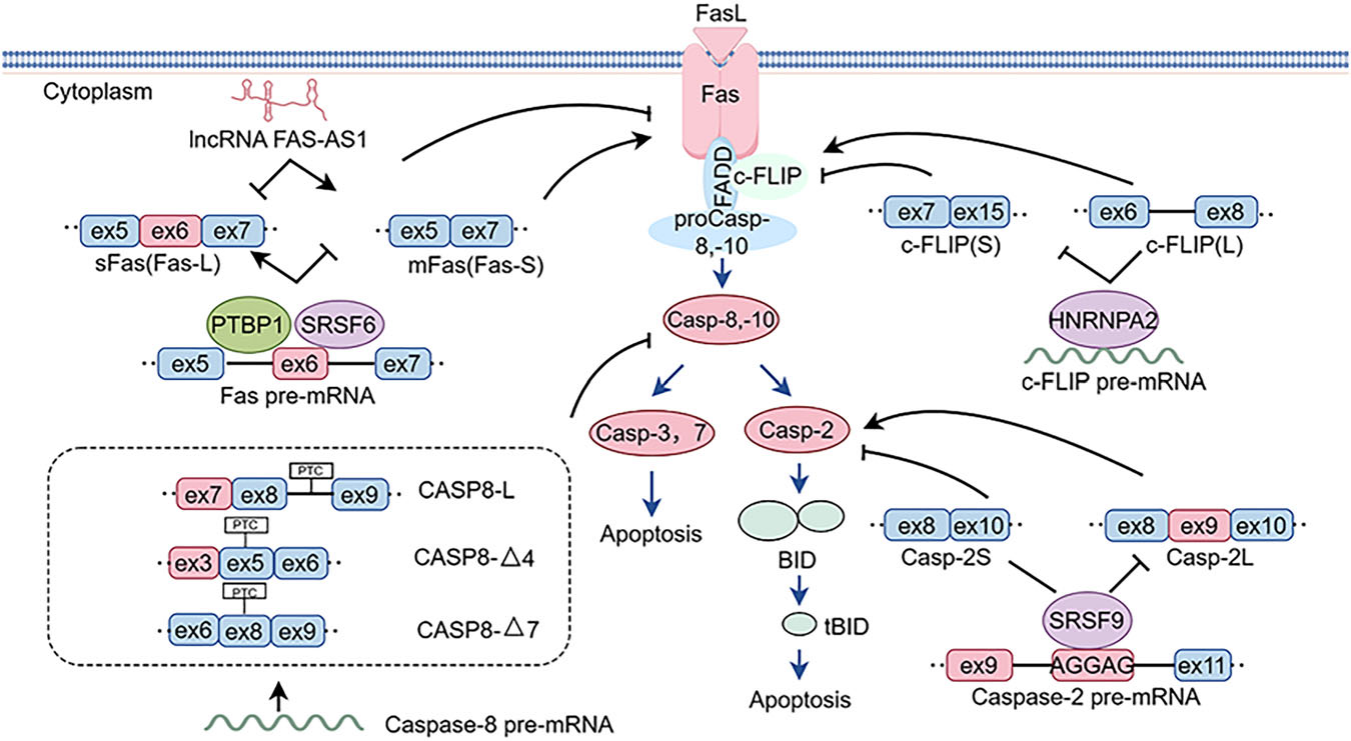
Mechanism diagram of the extrinsic apoptosis pathway regulated by alternative splicing. The extrinsic apoptosis pathway is a form of regulated cell death initiated by the self-catalysis and activation of procaspase-8 mediated by extracellular ligands - transmembrane receptors. Splicing factors including PTBP1, SRSF6, and HNRNPA2 activate Casp-8 to initiate apoptosis, and SRSF9 activates Casp-2 to cleave BID, promoting the release of Cyto c and apoptosis. Tumour cells usually utilise the AS mechanism mediated by splicing factors to evade apoptosis and acquire resistance to chemotherapy. Targeting these splicing factors and splicing variants in the apoptosis pathway may enhance the therapeutic effect of chemotherapy in drug-resistant cancers.

**Fig. 3:**
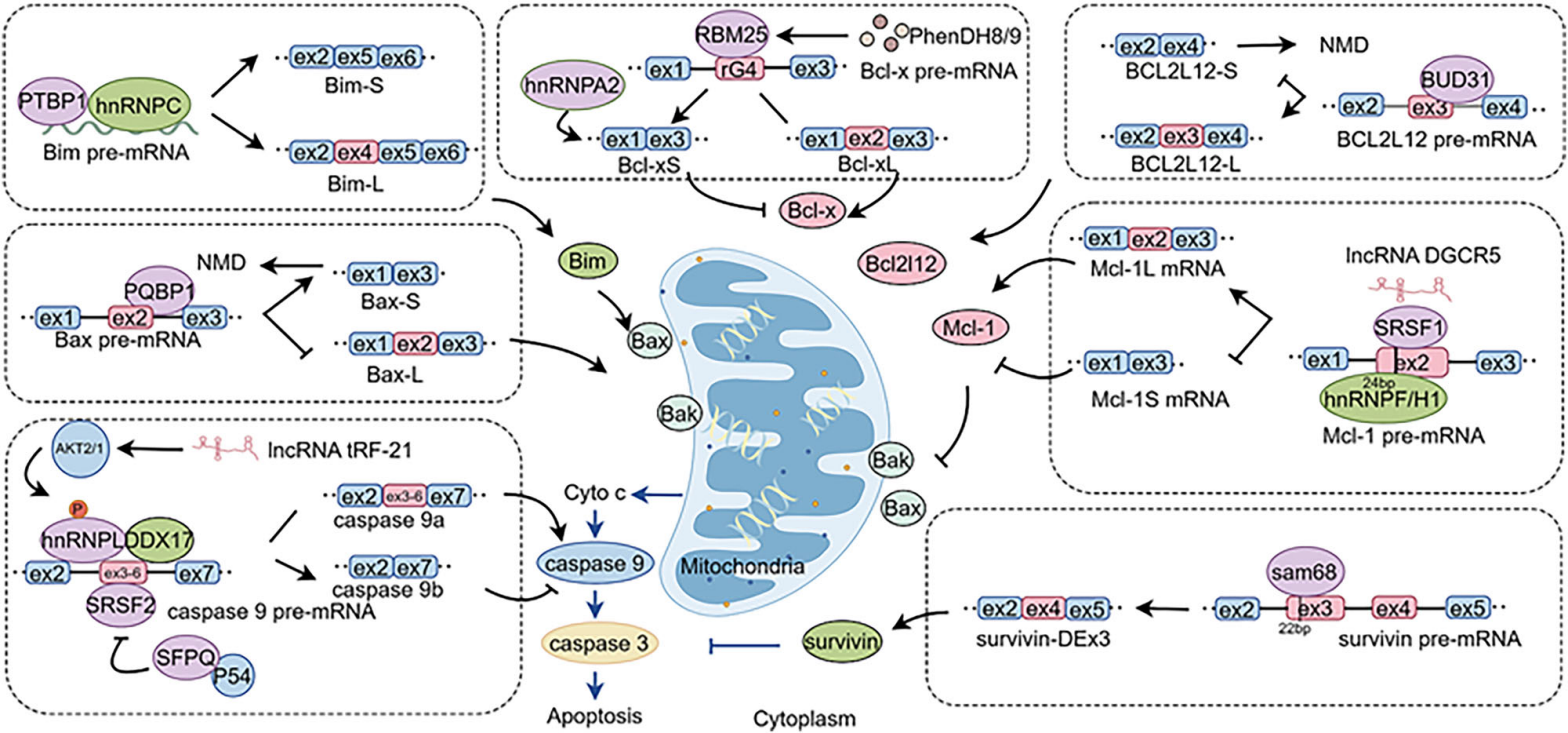
Regulation mechanism of splicing factors on the intrinsic apoptosis pathway. Intrinsic apoptosis is a regulatory cell death pathway mediated by mitochondria, triggered by stress stimuli such as DNA damage, which leads to the release of Cyto c and the activation of Caspase 9. Splicing factors exert their effects by regulating Caspase-9 and various key regulatory factors, including pro-apoptotic and anti-apoptotic factors. Splicing factors such as hnRNPL, DDX17, SRSF2, SFPQ, and sam68 regulate the activity of the Caspase family; splicing factors including PQBP1, PTBP1, and hnRNPC regulate pro-apoptotic factors, while splicing factors such as RBM25, hnRNPA2, BUD31, SRSF1, hnRNPF/H1 and the regulate the Cyto c release pathway and anti-apoptotic factors.

In the intrinsic pathway, cytochrome C is released from mitochondria into the cytoplasm in response to DNA damage and other stimuli and forms apoptotic bodies with caspase-9 and apoptotic peptidase activator 1 (Apaf-1) [26], ultimately activating caspase 3 to execute apoptosis [27]. BCL-2 protein family members, including the antiapoptotic proteins BCL-x, BCL2L12, BCL-2 and MCL-1 and the proapoptotic proteins Bim, Bax, Bak and Bid, regulate intrinsic apoptosis by controlling cytochrome C release [28, 29]. In addition, the IAP family, such as XIAPs and survivin, inhibits the activation of multiple caspases and negatively regulates apoptosis [30]. AS of apoptosis-related genes is an important regulatory event of apoptosis. The pre-mRNAs of these genes, through AS, generate multiple transcripts encoding proteins with different apoptotic functions. The AS events and splicing factors involved in regulating extrinsic and intrinsic apoptosis are shown in Fig. 2 and Fig. 3, respectively.

### The extrinsic pathway

#### Fas receptor

The Fas receptor is a transmembrane death receptor encoded by the *Fas* gene and belongs to the tumour necrosis factor (TNF) family [31]. The AS of exon 6 of *Fas* pre-mRNA results in the production of a membrane-bound isoform (mFas, exon 6 retained) that promotes apoptosis and a soluble isoform (sFas, exon 6 skipped) that inhibits apoptosis [32] (Fig. 2, mFas, sFas). It has a regulatory effect on the occurrence and development of various tumour types, such as breast cancer [33], nasopharyngeal carcinoma [34], and hepatocellular carcinoma (HCC) [35].

Zhen Guo et al. reported that the lncRNA *FAS-AS1*, whose expression is low in nasopharyngeal carcinoma cells, can regulate *Fas* AS. Overexpression of this gene increases the expression of mFas, decreases the expression of sFas, and promotes apoptosis [34]. However, the specific mechanism is unclear. Hepatitis B virus (HBV) infection is among the high-risk factors for HCC. HBV core protein can increase the expression of PTBP1, and PTBP1 binds ESS on exon 6 to increase the expression of sFas and reduce the expression of mFas, leading to inhibition of apoptosis and carcinogenesis [35]. Moreover, SRSF6 modulates the AS of the *Fas* gene in colorectal cancer (CRC) by binding to the UGCCA sequence in exon 6, increasing exon 6 inclusion, and this regulation requires a 5’ splice site but not a 3’ splice site [36].

#### c-FLIP

The AS of *c-FLIP* generates many transcripts, among which only two encode proteins: c-FLIP_S_ (without exons 8–14) and c-FLIP_L_ (without exon 7) (Fig. 2, c-FLIP_S_, c-FLIP_L_); c-FLIP_S_ negatively regulates apoptosis, while c-FLIP_L_ stimulates caspase-8 activation and apoptosis [37]. Apigenin can interact with the splicing factor hnRNPA2 to regulate *c-FLIP* splicing and reduce c-FLIP protein levels, leading to the apoptosis of lung cancer cells [38].

#### Caspase 8

Caspase 8 is a key proteolytic enzyme in the apoptotic cascade [39]. In adult T-cell leukaemia cells, AS of intron 8, exon 4 and exon 7 of *caspase 8* pre-mRNA produces three variants: CASP8L, CASP8-ΔE4 and CASP8-ΔE7 (Fig. 2, CASP8L, CASP8-ΔE4, CASP8-ΔE7). The retention of intron 8 in CASP8L introduced a premature stop codon (PTC), and the exon 4-skipped variant CASP8-ΔE4 introduced PTC in exon 5, which was the same as the exon 7-skipped variant CASP8-ΔE7. Thus, these variants lack the caspase catalytic domain encoded by exons 9 and 10 and exhibit a lower ability to induce apoptosis. Moreover, these variants bind to wild-type caspase 8, inhibiting its homodimerization and further inhibiting apoptosis [40].

#### Caspase 2

Caspase 2 is the most evolutionarily conserved member of the mammalian caspase family [41]. *Caspase 2* pre-mRNA generates a pro-apoptotic long mRNA (Casp-2L, with exon 9 retained) and an anti-apoptotic short mRNA isoform (Casp-2S, with exon 9 skipped) via AS (Fig. 2, Casp-2L, Casp-2S) [42]. Studies have shown that SRSF9 can bind to the AGGAG sequence in exon 10 in HeLa cells, modulate the AS of exon 9, and reduce the expression of Casp-2L [43].

### The intrinsic pathway

#### Caspase 9

Caspase 9, a pivotal player in the mitochondrial pathway, can undergo AS to generate two isoforms: proapoptotic caspase 9a and antiapoptotic caspase 9b (Fig. 3, caspase 9a, caspase 9b) [44]. Unlike caspase 9a, caspase 9b lacks the central catalytic domain encoded by exons 3–6 and has no cysteine protease activity. It attaches to apoptotic bodies and inhibits the cascade of caspase enzymes [45]. Caspase 9b can promote the progression of lung cancer [44].

Noncoding RNA transfer RNA-derived fragment 21 (*tRF-21*) is a suppressor of pancreatic ductal adenocarcinoma (PDAC) and inhibits AKT2-mediated phosphorylation of hnRNPL. Dephosphorylated hnRNPL could not interact with DDX17 to form a splicing complex, which reduced the splicing of *caspase 9* pre-mRNA to form caspase 9a and ultimately inhibited apoptosis and malignant phenotypes [46]. In epithelial ovarian cancer (EOC), SFPQ forms a complex with p54 to inhibit the binding of SRSF2 to *caspase 9* pre-mRNA, promote the expression of caspase 9b, and inhibit platinum-induced apoptosis, leading to chemoresistance [47].

#### BCL-X

The AS of *Bcl-x* exon 2 produces two functionally distinct isoforms, namely, Bcl-xL (exon 2 retained), which has an antiapoptotic effect, and proapoptotic Bcl-xS (exon 2 skipped) (Fig. 3, Bcl-xL, Bcl-xS), and the high expression of Bcl-xL is an important reason for tumour cell drug resistance [48].

Recent studies have revealed that the splicing factor RBM25 controls the AS of *Bcl-x* and promotes the production of BCL-xS in lung cancer cells. RBM25 specifically binds to the RNA G-quadruplex (rG4) around the 5’ splice site in exon 2, and this interaction is dependent on the arginine-rich glutamate motif of RBM25. Interestingly, two rG4 ligands, PhenDH8 and PhenDH9, were shown to increase the binding of RBM25 to rG4, promoting BCL-xS expression and apoptosis. These two small molecular compounds may be used to reverse drug resistance in lung cancer cells in the future [49]. In another study, Tshepiso Jan Makhafola et al. reported that the extract of Cotyledon orbiculata regulated *hnRNPA2/B1* AS, causing the hnRNPB1 isoform to switch to the hnRNPA2 isoform, and that hnRNPA2 further upregulated the expression of BCL-xS through AS, leading to the apoptosis of CRC and oesophageal cancer cells [50].

#### BCL2L12

BCL2L12 is a member of the antiapoptotic BCL2 family. The classical BCL2L12 protein contains a highly conserved BH2 domain, BH3-like motifs and a proline-rich region [51]. The two isoforms generated by AS include complete BCL2L12-L and the truncated BCL2L12-S, whose RNA sequences differ because of the skipping of exon 3 (Fig. 3, BCL2L12-L, BCL2L12-S) [52].

The spliceosome component BUD31 binds to the junction of exon 3 and intron 3, promotes the inclusion of exon 3 to produce BCL2L12-L, and enhances the malignant phenotype of ovarian cancer. However, knockdown of *BUD31* or splice-switching oligonucleotides (SSOs) promoted exon 3 skipping and caused BCL2L12-S to undergo nonsense-mediated degradation (NMD), leading to apoptosis [52].

#### Bax

Bax, a member of the BCl2 family, is a mitochondrial pore-forming protein [53]. Its variants, Bax-S and Bax-L, are produced mainly by the AS of exon 2 (Fig. 3, Bax-S, Bax-L). The splicing factor PQBP1 binds to exon 2 and intron 2, promoting the skipping of this exon to generate the Bax-S isoform, which introduces PTC because of the lack of exon 2; it is eventually degraded by NMD, thus making ovarian cancer cells resistant to apoptosis. However, the knockdown of *PQBP1* or the increase in exon 2 inclusion with SSO could increase the expression of Bax-L and inhibit ovarian cancer cell growth [54]. Interestingly, in CRC cells, the skipping of exon 2 and the deletion of microsatellite guanine in exon 3 restored the reading frame of this transcript, generating a new proapoptotic isoform, Bax∆2, which multimerizes as a platform to recruit and activate caspase 8 to trigger apoptosis [55, 56].

#### Bim

Bim can directly activate Bax/Bak [57]; it can also activate Bax/Bak by inhibiting BCL-2 and MCL-1 expression and ultimately activate intrinsic apoptosis [58]. Bim activity is determined by the AS of exons 3 and 4, which produce three protein variants: BimS, BimL, and BimEL. BimEL contains exons 3 and 4, BimL lacks exon 3, and BimS lacks two exons (Fig. 3, BimS, BimL). Among them, BimS has the strongest proapoptotic ability, whereas BimEL has the weakest [59]. In chronic myelogenous leukaemia (CML) cells, PTBP1 and hnRNP C promote exon 3 skipping to produce more proapoptotic variants BimS and BimL. However, knockdown of *PTBP1*, but not *hnRNP C*, inhibited Bim-mediated apoptosis [60]. Similarly, knockdown of *SRSF2* reduced BimS expression and inhibited intrinsic apoptosis in RCC [61].

#### Mcl-1

Mcl-1 overexpression is associated with poor prognosis and drug resistance in many cancers, and it regulates the mitochondrial apoptosis pathway [62]. AS of exon 2 of the *Mcl-1* gene produces two functionally distinct proteins, namely, antiapoptotic Mcl-1L (exon 2 retained) and proapoptotic Mcl-1S (exon 2 skipped) (Fig. 3, MCL-1L, MCL-1S) [63].

hnRNPF, H1 and K regulate *Mcl-1* splicing in breast cancer cells through direct binding to *Mcl-1* pre-mRNA. Among them, hnRNPF and hnRNPH1 bound ISE sequences only 24 bp from the 5’ splice site to promote exon 2 retention and Mcl-1L isoform expression. Simultaneous knockdown of these genes activates caspase 9-mediated apoptosis [64]. LncRNA *DGCR5*, which is highly expressed in oesophageal squamous cell carcinoma (ESCC), binds to SRSF1 to increase its protein stability. SRSF1 modulates the AS of *Mcl-1*, increases the expression of the Mcl-1L isoform, and increases cell resistance to apoptosis [65].

#### Survivin

Survivin, encoded by the *BIRC5* gene, is antiapoptotic in a variety of tumours, such as breast cancer and renal cell carcinoma [66]. Its antiapoptotic function is mediated in part by the inhibition of caspase 3 activity. *BIRC5* AS generates five functional variants, antiapoptotic-survivin and pro-apoptotic-survivin 2α (deletion of exons 3 and 4 and retention of the 3’ UTR), proapoptotic-survivin 2β (partial intron 2 retention), antiapoptotic-survivin 3β (partial intron 3 retention), and antiapoptotic-survivin DEx3 (lack of exon 3) [67, 68]. Sam68 binds to the 22 bp ESE on exon 3, promoting exon skipping and resulting in high expression of the survivin Dex3 isoform in breast and cervical cancer cells (Fig. 3, survivin Dex3) [68]. In addition, recent studies have revealed that survivin 2β enhances gemcitabine resistance in patients with PDAC [69].

## AS involved in regulating necroptosis

Necroptosis is a process of cellular self-destruction activated by extracellular signals (death receptor–ligand binding) or intracellular signals (foreign microbial nucleic acids) when apoptosis is blocked [70]. For example, in TNF-mediated necroptosis, after TNF binds to TNF receptor 1 (TNFR1), TNFR1 recruits multiple proteins to form complex I. Then, owing to the internalisation of TNFR1, complex II containing RIPK1, caspase 8 and c-FLIP is formed and activates caspase 8. When the activity of caspase 8 is blocked by pharmaceutical inhibitors or genetic intervention, RIPK1 and RIPK3 aggregate into a complex referred to as a necrosome, where mixed lineage kinase domain-like pseudokinase (MLKL) is phosphorylated by RIPK3 [71]. This phosphorylation event causes MLKL to form a complex at the plasma membrane, leading to cell swelling, membrane rupture and ultimately necroptosis. Dysregulation of necroptosis can lead to various human diseases, including cancer, and it has recently emerged as an important event in the regulation of tumorigenesis [72]. Nevertheless, two roles for necroptosis in cancer have been identified. Under some conditions, combinations of one or more necroptosis inhibitors may increase cancer progression and metastasis, and in other conditions, they may prevent tumour formation and metastasis [73, 74].

### MLKL

AS of microexon 10 of *MLKL* generates 2 isoforms: MLKL1 (microexon 10 skipped) and MLKL0 (microexon 10 retained) in human CRC and lung cancer cells. MLKL1 is pronenecroptotic, and protein structure analysis indicated that the C-terminal α-helix of MLKL1 is accommodated into a hydrophobic groove and that this interaction is essential for MLKL1 activation. The small molecular inhibitor MBA-h1 could target this interaction and block necroptosis. MLKL0 interacts with MLKL1 and RIPK3 and inhibits their activation and necroptosis [75].

### c-FLIP

Some studies have shown that the AS of *c-FLIP* is crucial for cell necroptosis and can modulate complex II; thus, in addition to its role in apoptosis, c-FLIP also plays a role in necrosis [76]. The c-FLIP isoform in complex II determines whether cell death is caused by RIPK3-dependent necroptosis or caspase-dependent apoptosis [77]. c-FLIP_L_ prevents the formation and stability of complex II, whereas c-FLIP_S_ promotes its assembly and blocks caspase 8 activity; subsequently, RIPK1 forms necrosomes with RIPK3 and activates MLKL, leading to necroptosis.

### NF-Y

Recently, another investigation revealed a relationship between AS and necroptosis in human neuroblastoma (NB). NF-Y is a heterotrimer transcription factor composed of NF-YA, NF-YB, and NF-YC subunits, all of which are required for DNA binding and transcriptional activity [78]. The *NF-YA* gene is divided into 9 exons, and a novel splice variant, NF-YAx, was identified in NB. Compared with the full-length variant, the NF-YAx is characterised by the skipping of exons 2, 4, and 6. NF-YAx can induce necroptosis and inhibit colony formation, but the detailed mechanism remains unclear [79].

## AS involved in regulating pyroptosis

Pyroptosis, also known as inflammatory necrosis, is a type of RCD that manifests as the continuous swelling of cells until the cell membrane ruptures, resulting in the release of cell contents and the activation of a strong inflammatory response [80]. Pyroptosis is an important natural immune response and plays an important role in fighting infection. It is carried out by the gasdermin (GSDM) family; the human GSDM family includes six members, GSDM A/B/C/D/E and DFNB59, among which the N-terminal fragment of GSDM A/B/C/D/E has membrane perforation activity and the C-terminus has autoinhibitory activity. In the presence of exogenous microbes or endogenous stimulation, active caspase 1/3/4/5/8/9/11 or granzyme A/B cleaves GSDMs and releases N-terminal fragments, which oligomerize and form pores in the cell membrane to release inflammatory molecules and induce pyroptosis [81]. Recently, some studies have shown that pyroptosis can affect tumour proliferation, invasion and metastasis [82].

### Signal transducer and activator of transcription (STAT3)

STAT3 is a critical component of the JAK/STAT signalling pathway, and its activation is involved in many cancers [83]. Interestingly, the AS of *STAT3* has been implicated in pyroptosis. STAT3 has 2 variants, STAT3α and STAT3β, produced by the AS of exon 23. STAT3β differs from STAT3α in that the deletion of 55 amino acid residues at the C-terminus results in a dominant negative effect because of the lack of a transcriptional activation domain [84]. STAT3β can localise to the mitochondria and inhibit the phosphorylation of STAT3α at Ser727 through competition for ERK1/2 binding, leading to disruption of the electron transport chain and high levels of ROS. High levels of ROS activate caspase 3, which cleaves GSDME and enhances pyroptosis in ESCC cells [85].

### GSDMB

GSDMB, one of the most important effector proteins of pyroptosis, has at least five isoforms, GSDMB 1–5, produced by the AS of exon 6 and exon 7 [86, 87]. Recently, multiple studies have reported that different splicing isoforms of GSMDB have distinct pyroptotic activities. GSDMB 3 and 4, which contain exon 6, can undergo pyroptosis, but isoforms 1, 2, and 5 have a deleted or truncated exon 6 and cannot. Further investigation revealed that GSDMB splicing isoforms have different membrane perforation activities, and structural analysis revealed that 13 amino acid residues encoded by exon 6 form a β sheet and that this conserved structure is necessary for GSDM pore formation. Noncytotoxic GSDMB 1/2 isoforms can inhibit cytotoxic GSDMB 3/4-induced pyroptosis and are negative regulators of GSDMB pyroptosis [88]. Thus, tumours can use the noncytotoxic isoform to downregulate the pore-forming ability of the cytotoxic isoform. Under natural killer cell attack, GSDMB isoforms 3 and 4 induce pyroptosis and lead to tumour cell death [88, 89].

More importantly, studies by Qing Kong revealed that in primary tumours, lower GSDMB3/4 expression may be associated with better clinical outcomes and reduced tumour risk, whereas noncytotoxic GSDMB1/2 is often overexpressed in tumours and may play a role in promoting tumorigenesis. These findings suggest that tumours may avoid pyroptosis by altering the spliced form of GSDMB and that this AS event is a possible drug target [88]. Manipulating this AS event in tumour cells to upregulate the expression of toxic GSDMB isoforms may improve antitumour immune responses in the tumour microenvironment. However, to date, we know almost nothing about the regulation of this AS event in tumours, and it is worth studying how splicing factors or RNA-binding proteins manipulate this AS event to provide a more theoretical basis for drug development.

## AS involved in regulating ferroptosis

**Fig. 4:**
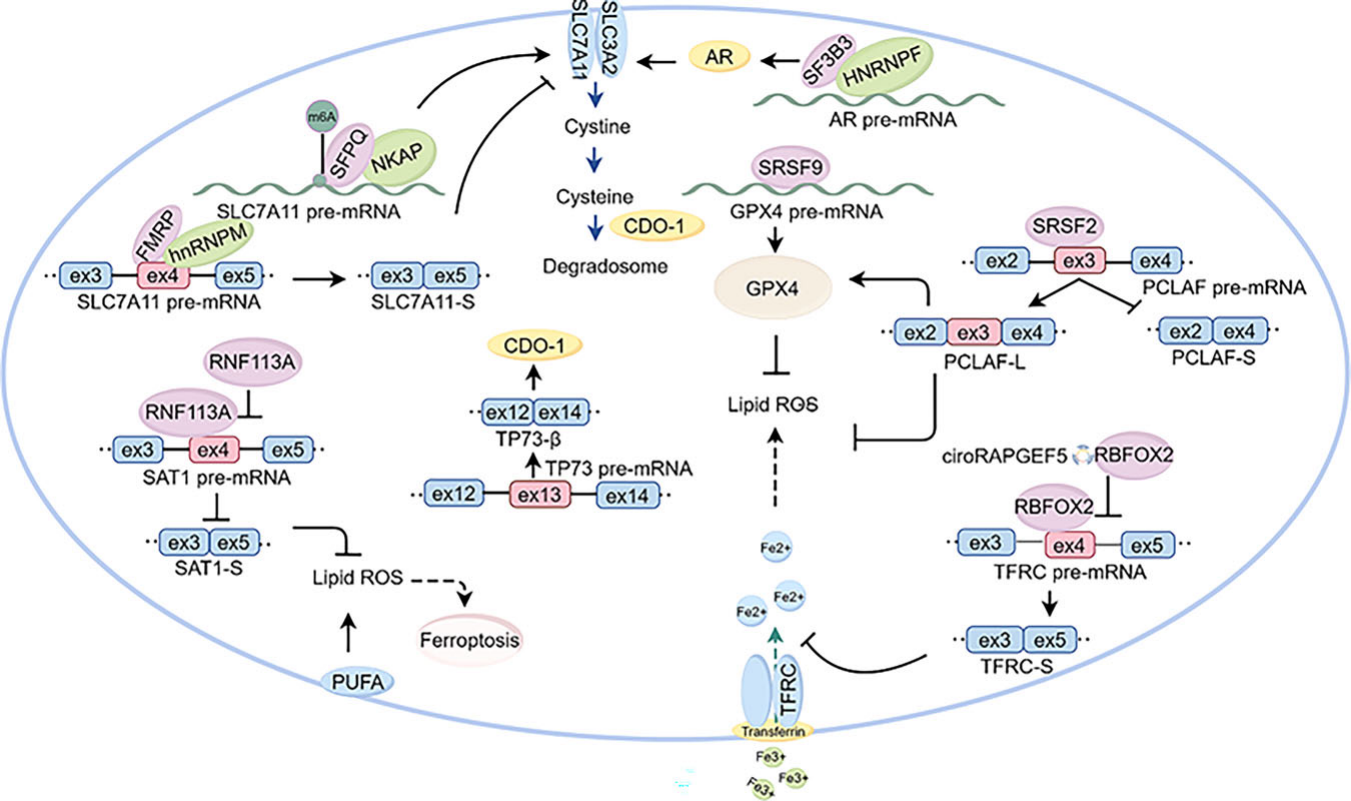
Splicing factors as critical regulators of ferroptosis. The unique iron-dependent regulatory cell death form of ferroptosis lipid peroxidation overproliferation is triggered by excessive lipid peroxidation and is regulated by multiple regulators. These splicing factors affect the susceptibility to ferroptosis by regulating glutathione biosynthesis (such as SFPQ, NKAP, FMRP, SF3B3, HNRNPF), GPX4 activity (SRSF2), and lipid peroxidation dynamics (such as RBFOX2, RNF113A), thereby determining the fate of tumour cells. Abnormal expression of splicing factors leads to the escape of ferroptosis.

Ferroptosis is a novel type of iron-dependent RCD that is distinct from apoptosis, necrosis, and autophagy. Ferroptosis is involved in a variety of biological metabolic and pathological processes in the body and is widely involved in the formation and development of malignant tumours [90]. Accumulating evidence indicates that aberrant ferroptosis is associated with aggressive cancer progression. Therefore, targeting ferroptosis has become a hot research area for cancer therapy [91]. The main mechanism of ferroptosis is that under the action of ferrous iron or ester oxygenase, the highly expressed unsaturated fatty acids on the cell membrane are catalysed, and lipid peroxidation occurs, thereby inducing cell death [92]. Cells have evolved numerous defence mechanisms to resist ferroptosis, such as glutathione peroxidase 4 (GPX4), which uses reduced glutathione (GSH) as a co-factor to reduce toxic lipid peroxides to lipid alcohols and ultimately inhibits ferroptosis [93]. GSH synthesis requires cysteine as the precursor, and intracellular cysteine is converted mainly from cystine, which is imported from the extracellular environment by the subunit solute carrier family 7 member 11 (SLC7A11)-mediated cystine transporter system xCT [94]. Thus, AS of these genes may modulate ferroptosis in tumour cells, the AS events and splicing factors involved in regulating ferroptosis are summarised in Fig. 4.

### SLC7A11

In glioblastoma (GBM) cells, the RNA binding protein (RBP) NF-κB-activating protein (NKAP) acts as a ferroptosis inhibitor by regulating the AS of *SLC7A11* through m6A. NKAP functions as a m6A reader and binds to the m6A site of *SLC7A11* pre-mRNA; then, it recruits the splicing factor proline and glutamine-rich (SFPQ) to recognise the splicing site and eventually facilitates retention of the last exon, ultimately leading to the upregulation of SLC7A11 expression [95]. Similarly, another m6A reader, fragile X mental retardation protein (FMRP), also modulates ferroptosis via the AS of *SLC7A11*. Highly expressed FMRP interacted with hnRNPM and promoted the skipping of exon 4 of *SLC7A11* pre-mRNA and expression of SLC7A11-S (exon 4 skipped) (Fig. 4, SLC7A11-L, SLC7A11-S). Compared with SLC7A11-L (exon 4 retained), SLC7A11-S conferred greater ferroptosis resistance to breast cancer cells [96].

### Transferrin receptor (TFRC)

Transferrin receptor (TFRC) encodes transferrin receptor protein 1, which reacts with transferrin to import extracellular iron and has been reported to promote cancer cell ferroptosis [97]. Interestingly, circRAPGEF5 can inhibit ferroptosis in endometrial cancer (EC) cells by modulating the AS of *TFRC*. Mechanistically, circRAPGEF5 interacts with the RNA binding protein fox-1 homologue 2 (RBFOX2) and obstructs the binding of RBFOX2 to *TFRC* pre-mRNA, leading to the skipping of exon 4 (Fig. 4, TFRC-S). The protein translated by this truncated isoform cannot localise to the cell membrane and fails to import iron, ultimately conferring ferroptosis resistance in EC cells [98].

### Spermidine/spermine N1-acetyltransferase 1 (SAT1)

SAT1 is involved in polyamine catabolism, and its activation induces lipid peroxidation and ferroptosis in human tumours [99]. Some studies have reported that the RBP RNF113A modulates the splicing of *SAT1* to cause ferroptosis in lung cancer cells. The *SAT1* gene has 7 exons, among which exon 4 contains a PTC and needs to be skipped to generate the mRNA encoding a functional protein. Upon stimulation with cisplatin, RNF113A suppresses the skipping of exon 4 and subsequent *SAT1* expression, conferring ferroptosis resistance [100] (Fig. 4, SAT1-S).

### p73

p73, like p53, acts as a tumour suppressor by inducing the expression of an array of genes involved in growth suppression [101]. AS of exon 13 of *TP73* generates two isoforms, p73α and p73β, and p73β is relatively small and lacks a transcription-inhibiting domain; thus, it is considered to be the active isoform (Fig. 4, p73β). Jin Zhang used CRISPR to knockout *TP73* exon 13 and enhanced p73β expression in lung cancer cells. p73β transcriptionally increased cysteine dioxygenase 1 (CDO-1) expression, leading to intracellular cysteine depletion and ultimately accelerated ferroptosis [102].

### Proliferating cell nuclear antigen clamp-associated factor (PCLAF)

PCLAF is a regulator of DNA repair during DNA replication and is involved in many cancers. AS of exon 3 produces two variants, PCLAF-L (exon 3 retained) and PCLAF-S (exon 3 skipped) (Fig. 4, PCLAF-L, PCLAF-S), and PCLAF-L functions as an oncogene in HCC, whereas PCLAF-S does not [103]. Recent investigations revealed that HBV infection leads to sorafenib resistance in HCC through SRSF2-modulated *PCLAF* splicing. HBV suppresses the expression of SRSF2, which enhances the inclusion of exon 3 and increases the generation of the PCLAF-L isoform. Further research revealed that the SRSF2/PCLAF-L axis reduces intracellular Fe^2+^ levels and increases GPX4 expression, resulting in decreased ferroptosis. However, the detailed molecular mechanism of this regulation is unclear. These findings indicate that the SRSF2/PCLAF-L axis may be a prospective molecular therapeutic target for patients with sorafenib-resistant HBV-related HCC [103].

### Androgen receptor (AR)

Androgen deprivation therapy is important for the clinical management of prostate cancer; however, many patients eventually become resistant to these drugs, such as enzalutamide and abiraterone acetate, and develop castration-resistant prostate cancer (CRPC) [104]. Aberrant activation of AR signalling is believed to be the primary reason for castration resistance, and AS of *AR* can generate constitutively active forms of AR. The splicing factors HNRNPF and SF3B3 have been reported to regulate the AS of *AR* pre-mRNA to generate active AR (Fig. 4, AR) [105, 106]. Interestingly, the ferroptosis inducer erastin could suppress the expression of *AR* splice variants in CRPC and synergistically inhibit the growth of CRPC cells when combined with androgen deprivation therapy drugs [107]. Although the detailed molecular mechanism of this phenomenon is unclear, these findings suggest that erastin has the potential to increase the synergy of androgen deprivation therapy.

### GPX4

An investigation by Rui Wang revealed that SRSF9 is involved in the regulation of ferroptosis in CRC. It can interact with *GPX4* mRNA and promote the protein expression of GPX4, ultimately inhibiting ferroptosis [108]. However, whether SRSF9 regulates GPX4 protein expression by manipulating the AS or stability of *GPX4* mRNA is unknown (Fig. 4, GPX4).

## AS involved in regulating autophagy

**Fig. 5:**
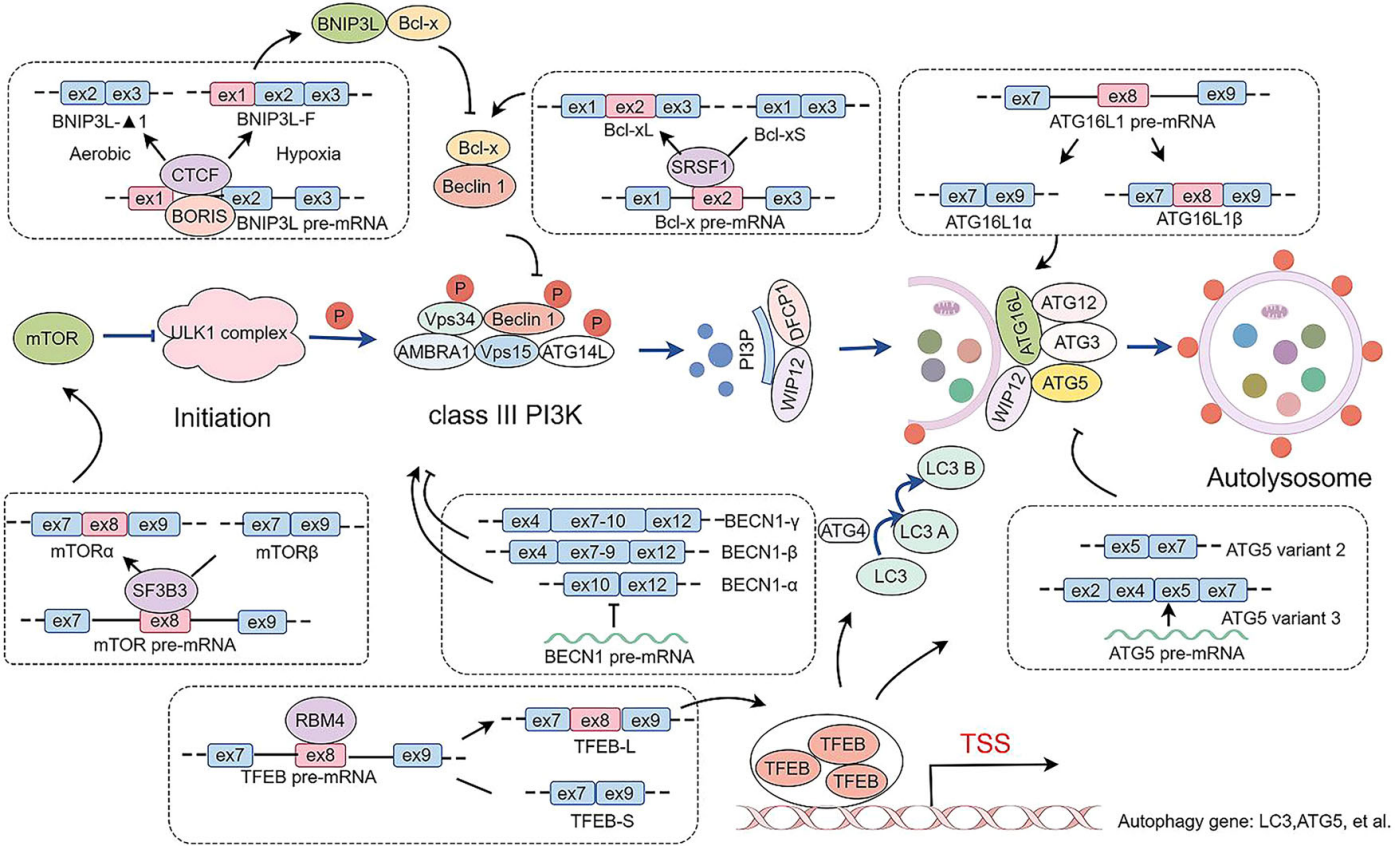
Splicing factors-driven modulation of autophagy in cancer cells. In tumour cells, splicing factors coordinate the various stages of autophagy. By regulating the components of the mTOR-controlled ULK1 complex (such as SF3B3), modulating the Beclin1-VPS34 binding complex (such as CTCF, BORIS, SRSF1), and indirectly regulating the Atg5-Atg12-Atg16L1 complex (RBM4), splicing factors affect the maturation of autolysosome.

Autophagy is a normal life process in which cells use lysosomes to degrade their own biological macromolecules and organelles and release small molecules for recycling [109]. This process requires the cooperative participation of a variety of proteins. First, the ULK1 complex phosphorylates the class III PI3K complex (including VPS34 and BECN1) to produce PI3P [110]. PI3P recruits WIPI2 and the Atg16L1–Atg5–Atg12 complex to the phagophore [111]. Moreover, the C-terminus of LC3 is cleaved by Atg4 to produce LC3A, which is further lipidated into LC3B under the action of the Atg16L1–Atg5–Atg12 complex, and LC3B is involved in the extension and maturation of autophagosomes [112]. Autophagy is regulated by mTOR signalling, and when the energy supply is sufficient, the phosphorylation of ULK1 by mTOR inhibits its activation and autophagy [113]. Like necroptosis, autophagy has two functions in tumours, and some antitumour strategies targeting autophagy have been developed [114]. Below, we discuss key splicing factor as regulators of autophagy (Fig. 5).

## mTOR

Persistent activation of the mTOR pathway contributes to the occurrence and development of many tumours. *mTOR* exon 8 retention yields the full-length functional isoform mTORα [115]. Tong Xu et al. reported that SF3B3 could regulate this exon AS in breast cancer, CRC, liver cancer and cervical cancer. SF3B3 silencing inhibited its retention and mTORα protein expression (Fig. 5, mTORα, mTORβ), relieved the inhibition of autophagy by mTOR signalling, and thus increased LC3B protein expression [115].

### BECN1

BECN1, as a key regulator of the class III PI3K complex, plays an important role in autophagy. In addition to full-length Beclin-1, multiple BECN1 variants have been identified in human cancer cells. BECN1 variant (lacking exons 10 and 11) was detected in both HeLa cells and CRC cells. BECN1s lose the ability to initiate autophagy but can activate mitophagy [116]. Exon 11 skipped variant BECN1-α, exons 5, 6, and 11 missed variant BECN1-γ, and exons 5, 6, 10, and 11 skipped variant BECN1-β and were cloned in breast cancer and ovarian cancer cells (Fig. 5, BECN1-α, BECN1-β, BECN1-γ). BECN1-α can activate autophagy, whereas BECN1-β inhibits autophagy, and BECN1-γ weakly regulates autophagy [117]. These findings suggest that cancer cells can utilise the AS of *BECN1* to regulate autophagy. However, the regulators of these splicing events are still unknown. SRSF3 can downregulate BECN1 expression, inhibit autophagy and promote tumorigenesis in oral squamous cell carcinoma (OSCC) cells [118], but whether this occurs through the regulation of AS needs further study.

### Atg16L1

In tumour cells, *Atg16L1* exon 8 AS generates two isoforms, ATG16L1α (exon 8 skipped) and ATG16L1β (exon 8 retained) (Fig. 5, ATG16L1α, ATG16L1β). Although both of them can mediate LC3 lipidation, Atg16L1β is not essential for autophagy and mainly catalyses LC3 lipidation on endosomes [119]. NSCLC cells regulate exon 8 AS to increase Atg16L1β expression, inhibit autophagy, and resist apoptosis and EGFR-TKI [120].

### Atg5

Dong–Yun Ouyang et al. reported that the prostate cancer cell line DU145 was unable to undergo autophagy, and further studies revealed that the cell expressed mainly variant 2 (lacking exon 6) and variant 3 (lacking exons 3 and 6), lacking full-length variant 1 (Fig. 5, ATG 5 variant 2, variant 3). However, variants 2 and 3 were unable to translate functional Atg5 protein because of the introduction of PTC, resulting in the failure of the AtG5–Atg12–Atg16L1 complex to form and activate autophagy [121].

### Bcl-x

The AS of *Bcl-x* also regulates autophagy in addition to apoptosis. In lung cancer cells, SRSF1 binds to *Bcl-x* pre-mRNA to stimulate the expression of Bcl-xL, and Bcl-xL binds to BECN1 to disrupt the formation of the BECN1-VPS34 complex and inhibit autophagy (Fig. 5, Bcl-xL, Bcl-xS). On the other hand, SRSF1 also directly binds to VPS34, disrupting the interaction between BECN1 and VPS34 and further inhibiting autophagy [122]. Moreover, SRSF1 is degraded through the autophagic pathway to activate autophagy, resulting in the formation of a positive feedback loop. The inhibition of SRSF1 expression and the activation of autophagy can inhibit the growth of gefitinib-resistant lung cancer cells both in vitro and in vivo, suggesting that the AS of *Bcl-x* is a potential target for tumour treatment.

### BNIP3L

*BNIP3L* is a hypoxia-induced autophagy gene. After binding to Bcl-xL, it inhibits the binding of BECN1 to Bcl-xL and promotes the entry of BECN1 into the class III PI3K complex to initiate autophagy. In breast cancer cells, *BNIP3L* exon 1 splicing produces two isoforms, BNIP3L-F (with exon 1) and BNIP3L-Δ1 (without exon 1) (Fig. 5, BNIP3L-F, BNIP3L-Δ1). The former stimulates autophagy, and the latter inhibits autophagy. Under hypoxic conditions, binding of CTCF to unmethylated *BNIP3L* intron 1 enhances exon 1 retention, increases BNIP3L-F expression, and activates autophagy. However, under aerobic conditions, BORIS binds to methylated *BNIP3L* intron 1 and recruits SRSF6 to bind to the *BNIP3L* 5′-UTR, inhibiting exon 1 retention and increasing BNIP3L-Δ1 expression to inactivate autophagy [123].

### Transcription factor EB (TFEB)

As a transcription factor, TFEB plays a critical role in autophagy regulation by binding to the promoters of autophagy-related genes (such as *LC3* and *ATG5*) and activating their expression [124]. In acute myeloid leukaemia (AML) cells, RBM4 binds to exon 8 of *TFEB* and promotes the retention of exon 8 to generate variant TFEB-L, which acts as an autophagic isoform and promotes cell differentiation (Fig. 5, TFEB-L, TFEB-S) [125].

## Targeting ASs involved in regulating RCD

Drugs that act on tumour cells through RCD, especially apoptosis, are among the treatment options for cancer. As an important regulatory modality, AS can regulate a variety of types of RCD, including apoptosis, autophagy, ferroptosis, pyroptosis, and necrosis, and can affect tumour development and treatment response. The interaction between AS and RCD provides us with new possibilities for the development of targeted therapy. Small-molecule drugs and inhibitors targeting splicing factors or SSOs targeting specific splicing events to activate or inhibit various RCDs can be used to treat tumours or reverse tumour resistance. These inhibitors and SSOs and their detailed information are presented in Table 2.

**Table 2:** Small molecule inhibitors and splice-switching oligonucleotides targeting alternative splicing-regulatory cell death axes in cancer.

Inhibitor or SSO	Target	Phase	Targeted cancers, experimental models and references
ABT-737	Bcl-xL, led to downregulation of Bcl-xL	Preclinical	The combination of ABT-737 and ruxolitinib promoted apoptosis and inhibited proliferation of myeloproliferative neoplasms cells [126]
E7107	SF3B, led to downregulation of Mcl-1L	I	Induced apoptosis of Mcl1-dependent NSCLC cells, inhibited NSCLC growth in vivo [139]
SACLAC	SF3B1, induced switch from anti-apoptotic Mcl-1L to pro-apoptotic Mcl-1S	Preclinical	Promoted AML cell apoptosis [127]
Auranofin	NONO, led to the retention of intron 1 of GPX1	Clinical	Promoted apoptosis of GBM cells and inhibited growth of GBM in mice [128].
Amiodarone	Potassium channels, induced retention of exon 4 of SRSF3 and degradation of SRSF3 pre-mRNA through NMD	Clinical	Promoted ROS-induced apoptosis of Hela cells [129]
SSO	Bcl-x, induced switch from anti-apoptotic Bcl-xL to pro-apoptotic Bcl-xS	Preclinical	Promoted apoptosis and death of melanoma cells, and inhibited metastasis of melanoma cells in mice [140] Promoted apoptosis and autophagy of GBM cells, and inhibited proliferation of GBM cells [141, 142]
	Bim, induced skipping of exon 3 and inclusion of exon 4, leading to upregulation of BimL	Preclinical	Promoted apoptosis of CML cells and resensitized CML cells to imatinib [143]
	SRSF3, induced inclusion of exon 4 and degradation of SRSF3 pre-mRNA through NMD	Preclinical	Promoted apoptosis and inhibited proliferation of OSCC cells [144, 145] Promoted apoptosis of breast cancer cells, enhanced therapeutic sensitivity of breast cancer cells to paclitaxel [145]

*NSCLC* non-small cell lung cancer, *AML* acute myelocytic leukaemia, *GBM* glioblastoma, *OSCC* oral squamous cell carcinoma, *CML* chronic myeloid leukaemia.

Ruxolitinib is clinically used for the treatment of myeloproliferative neoplasms (MPNs), but there are problems such as drug resistance and incomplete symptom relief. Bcl-xL, the antiapoptotic isoform produced by *Bcl-x* AS, is highly expressed in patients with myeloproliferative neoplasms. The combination of the Bcl-xL small-molecule inhibitor ABT-737 and ruxolitinib has synergistic effects, activating apoptosis and inhibiting cell proliferation [126]. The ceramide analogue SACLAC is an acid ceramidase inhibitor. Intriguingly, it can also kill AML cells via the modulation of AS. Mechanistically, it inhibits the expression of SF3B1, the core component of the spliceosome, changes the AS of *Mcl-1*, and transforms the antiapoptotic variant Mcl-1L into the proapoptotic variant Mcl-1S, thus inducing apoptosis [127]. The splicing factor NONO is highly expressed in GBM cells. It can bind to intron 1 of *GPX1* pre-mRNA and inhibit its retention. Auranovin, a small-molecule inhibitor of NONO, disrupted the regulation of splicing events by NONO, resulting in the retention of intron 1, failure to form normal *GPX1* transcripts, and inhibition of GPX1 expression. GPX1, an antioxidant enzyme, plays important roles in combating oxidative stress and maintaining redox balance. Decreased GPX1 expression leads to cellular ROS accumulation and apoptosis. Auranovin can inhibit the growth of GBM in a mouse model, which indicates that it may be used for the treatment of GBM in the future [128].

Chang YL et al. reported that the autophagy inducer amiodarone regulates the AS of exon 4 of *SRSF3*, promotes its retention and introduces PTC for degradation, thus reducing SRSF3 expression and increasing cell ROS-induced apoptosis [129]. Like in MPN, the expression of *Bcl-x* AS is aberrant in GBM, resulting in high expression of Bcl-xL. The use of SSO to increase the expression of Bcl-xS and reduce the expression of Bcl-xL can activate apoptosis on the one hand and relieve the inhibition of autophagy by BCL-XL on the other hand, and activated autophagy can also inhibit cell proliferation; these two processes work together to inhibit tumour progression and enhance the effect of radiotherapy [130].

## Conclusions and prospects

At both the individual level and the cellular level, cell death is inevitable for all living organisms and plays a crucial role in maintaining tissue homoeostasis and biological functions. There are various types of cell death, which can be generally classified into accidental cell death and RCD [131], and the latter is a complex and interrelated process. Common forms of RCD include apoptosis, necroptosis, ferroptosis, pyroptosis, and autophagy, each with unique molecular mechanisms and morphological characteristics [132]. In addition, these death modes are regulated by different biological macromolecules, and some molecules engage in “cross-talk” among different types of RCD. For instance, Beclin 1 not only serves as a caspase substrate in the apoptosis pathway but also participates in the formation of autophagosome membranes [133]. When the balance between cell proliferation and cell death is broken, cancer is triggered, and one of the hallmarks of cancer is the evasion of cell death. Therefore, dysregulation of cell death is a critical feature of cancer occurrence, and the mechanisms underlying the modulation of different RCDs provide potential therapeutic targets for cancer treatment.

AS is a ubiquitous posttranscriptional regulatory mechanism in eukaryotes through which proteins with various structures and functions are produced from a single pre-mRNA. These protein isoforms greatly enrich the proteome within the cell. The types of AS generally include exon skipping, intron inclusion, 5’ AS, 3’ AS, and mutual exclusion of exons. Compared with adjacent normal tissues, most cancer tissues exhibit more extensive aberrant splicing, and many studies have revealed that when the splicing of cell death-related genes is abnormal, it can lead to cancer. Splicing factors, such as those in the SRSF and hnRNP families, act as trans-acting factors and regulate splicing through interactions with splicing enhancers or silencers. However, the regulatory mechanisms of these two families are rather complex and not static. For example, PTBP1 is a classic repressor but can also activate splicing. Therefore, both SRSFs and hnRNPs can act as oncogenes to promote cancer occurrence or as tumour suppressors to inhibit cancer progression. This article reviews in detail the mechanisms through which these splicing factors and spliceosome components regulate the AS of RCD-related genes in recent years and summarises some related inhibitors.

Currently, many inhibitors targeting AS are entering clinical trials. Among them, personalised SSOs are powerful therapeutic tools. SSOs act on RNA degradation, AS changes and translation inhibition through the principle of base pairing. For instance, SSO is used to modulate the splicing of *PKM* from the PKM2 isoform to the PKM1 isoform, leading to HCC cell death [134]. SSO drugs targeting short *COX11* transcripts can effectively degrade mRNA, disrupt copper homoeostasis, and inhibit the development of gastric cancer [135]. To date, approximately 10 different SSO clinical drugs have been approved by the FDA for the treatment of neurological disorders and other genetic diseases [136, 137]. However, no SSO has been approved for clinical cancer treatment. Many studies have shown that targeting RCD-related splicing events with SSO can inhibit tumour cell growth both in vivo and in vitro. However, the clinical application of SSO drugs faces many challenges, such as hepatotoxicity and nephrotoxicity, as well as its instability as a nucleic acid molecule and difficulties in delivery, which make it prone to degradation and unable to cross barriers [138]. Future research can combine bifunctional ionic materials and nanomaterials with SSO to address their existing issues.

In addition to the direct killing of tumour cells by drugs to inhibit cancer, the tumour microenvironment has become a research hotspot in recent years. In cancer cells, continuous and dynamic interactions with the tumour microenvironment are crucial for tumorigenesis. This process involves immune cells, fibroblasts, the extracellular matrix, cytokines and many other noncancer cell components. Research on the tumour microenvironment has led to the development of cancer immunotherapies, including checkpoint inhibitors and tumour vaccines. Tumour cells can escape cell pyroptosis caused by cell death through the regulation of the AS of *GSDMB*. This discovery highlights the interaction and molecular mechanism between RCD regulated by AS and the tumour microenvironment, which can provide targets for developing treatments to improve the response rate or efficacy of tumour immunotherapy.

The pathogenesis of cancer is very complex. This review summarises the molecular mechanisms by which AS regulates various RCDs, which can help us better understand the aetiology of tumours. The clinical transformation of the targets identified in these studies, such as the development of precise and effective small-molecule inhibitors or SSO drugs, may solve some of the current challenges in clinical cancer treatment, improve treatment efficacy and benefit more cancer patients.

## Data Availability

The datasets generated/analysed during the current review are available.
